# Maraviroc Intensification of cART in Patients with Suboptimal Immunological Recovery: A 48-Week, Placebo-Controlled Randomized Trial

**DOI:** 10.1371/journal.pone.0132430

**Published:** 2015-07-24

**Authors:** Steven F. L. van Lelyveld, Julia Drylewicz, Maaike Krikke, Ellen M. Veel, Sigrid A. Otto, Clemens Richter, Robin Soetekouw, Jan M. Prins, Kees Brinkman, Jan Willem Mulder, Frank Kroon, Ananja Middel, Jori Symons, Annemarie M. J. Wensing, Monique Nijhuis, José A. M. Borghans, Kiki Tesselaar, Andy I. M. Hoepelman, MIRS study group

**Affiliations:** 1 Department of Internal Medicine & Infectious Diseases, University Medical Center Utrecht, Utrecht, The Netherlands; 2 Department of Immunology, University Medical Center Utrecht, Utrecht, The Netherlands; 3 Department of Internal Medicine, Rijnstate Hospital, Arnhem, The Netherlands; 4 Department of Internal Medicine & Gastroenterology, Spaarne Gasthuis, Haarlem, The Netherlands; 5 Department of Internal Medicine, Division of Infectious Diseases, Center for Infection and Immunity, Academic Medical Center, University of Amsterdam, Amsterdam, The Netherlands; 6 Department of Internal Medicine, Onze Lieve Vrouwe Gasthuis, Amsterdam, The Netherlands; 7 Department of Internal Medicine, Slotervaart Hospital, Amsterdam, The Netherlands; 8 Department of Infectious Diseases, Leiden University Medical Center, Leiden, The Netherlands; 9 Department of Medical Microbiology, University Medical Center Utrecht, Utrecht, The Netherlands; Faculty of Medicine, AUSTRALIA

## Abstract

**Objective:**

The immunomodulatory effects of the CCR5-antagonist maraviroc might be beneficial in patients with a suboptimal immunological response, but results of different cART (combination antiretroviral therapy) intensification studies are conflicting. Therefore, we performed a 48-week placebo-controlled trial to determine the effect of maraviroc intensification on CD4^+^ T-cell counts and immune activation in these patients.

**Design:**

Double-blind, placebo-controlled, randomized trial.

**Methods:**

Major inclusion criteria were 1. CD4^+^ T-cell count <350 cells/μL while at least two years on cART or CD4^+^ T-cell count <200 cells/μL while at least one year on cART, and 2. viral suppression for at least the previous 6 months. HIV-infected patients were randomized to add maraviroc (41 patients) or placebo (44 patients) to their cART regimen for 48 weeks. Changes in CD4^+^ T-cell counts (primary endpoint) and other immunological parameters were modeled using linear mixed effects models.

**Results:**

No significant differences for the modelled increase in CD4^+^ T-cell count (placebo 15.3 CD4^+^ T cells/μL (95% confidence interval (CI) [1.0, 29.5] versus maraviroc arm 22.9 CD4^+^ T cells/μL (95% CI [7.4, 38.5] p = 0.51) or alterations in the expression of markers for T-cell activation, proliferation and microbial translocation were found between the arms. However, maraviroc intensification did increase the percentage of CCR5 expressing CD4^+^ and CD8^+^ T-cells, and the plasma levels of the CCR5 ligand MIP-1β. In contrast, the percentage of *ex-vivo* apoptotic CD8^+^ and CD4^+^ T-cells decreased in the maraviroc arm.

**Conclusions:**

Maraviroc intensification of cART did not increase CD4^+^ T-cell restoration or decrease immune activation as compared to placebo. However, *ex-vivo* T-cell apoptosis was decreased in the maraviroc arm.

**Trial Registration:**

ClinicalTrials.gov NCT00875368

## Introduction

Treatment of HIV-infection with combination antiretroviral therapy (cART) suppresses viral replication, leading to recovery of CD4^+^ T cells. Unfortunately, 10–30% of the patients treated with cART experience a suboptimal immunological response, i.e. failure to restore CD4^+^ T-cell counts despite successful virological suppression [1–6]. Several studies have shown a worse long term clinical outcome in terms of death, AIDS and non-AIDS defining diseases in these patients [1,2,4,7,8].

The CCR5-antagonist maraviroc (MVC) was registered in 2008 for the treatment of antiretroviral treatment-naive (USA only) and -experienced HIV-1 infected patients [9]. Next to its established efficacy in suppressing plasma HIV-RNA, there has been much interest in the potential immunological effects of CCR5 antagonists. Molecular studies have shown that the CCR5 pathway can influence T-cell trafficking, activation and apoptosis [10–12]. In line with these observations, genetic studies have shown that the CCL3L1-CCR5 genotype influences the degree of CD4^+^ T-cell reconstitution during cART [13], and it was therefore postulated that manipulation of this pathway might enhance CD4^+^ T-cell recovery. Indeed, MVC containing regimens have been shown to lead to a larger increase in CD4^+^ T-cell counts than efavirenz containing regimens in treatment-naive HIV-1 patients [14]. A meta-regression analysis of clinical trials investigating the effects of CCR5-antagonists in antiretroviral treatment-experienced patients showed that the use of a CCR5-antagonist was associated with a significant additional increase in CD4^+^ T-cell counts of +30 (95% confidence interval (CI), 19–42) cells/μL in 24 weeks, independent of virological suppression [15]. Most recently, treatment with MVC containing regimens was shown to normalize regulatory T cell (Treg) numbers and frequency after 48 weeks of treatment [16].

In light of these findings intensification of cART with MVC might be of special interest for patients with a suboptimal immunological response despite adequate virological control and various intensification studies have been performed [17–23]. However the results of these studies are conflicting, and to date only one placebo-controlled intensification study of patients with a suboptimal immunological response on cART has been published [20]. Therefore, we performed the ‘Maraviroc Immune Recovery Study (MIRS), a 48-week, double-blind, placebo-controlled trial to study the effect of MVC intensification of cART on CD4^+^ T-cell recovery in HIV-1 infected patients.

## Methods

### Subjects

HIV-infected patients were recruited from 10 HIV treatment centers in the Netherlands. This study was approved on November 19, 2008, by the Ethical Committee of the University Medical Center Utrecht and all subjects provided written informed consent in accordance with the Declaration of Helsinki (EudraCT number 2008-003635-20). This study was registered on December 15, 2008, at the Netherlands Trial Registry (NTR1592), and additionally at ClinicalTrials.gov on April 1, 2009 (NCT00875368). The authors confirm that all ongoing and related trials for this drug/intervention are registered. Inclusion criteria were: age 18 years and older; a CD4^+^ T-cell count <350 cells/μL while at least two years on cART, or a CD4^+^ T-cell count <200 cells/μL while at least one year on cART; viral suppression (plasma HIV-RNA < 50 copies/ml) for at least 6 months prior to inclusion. Exclusion criteria were: previous use of MVC; HIV-2 infection; cART regimen containing a combination of tenofovir and didanosine; active infection treated with antimicrobial therapy; acute hepatitis B or C infection; and, radiotherapy or chemotherapy in the previous 2 years. The first patient was screened on February 19, 2009, whereas the final study visit of the last study participant took place on December 22, 2011.

### Study procedures

Included patients were randomized to add MVC or placebo to their existing cART regimen for 48 weeks. Randomization was performed by the pharmacy at the central site using specialized software, all study-personnel and study subjects were blinded during the entire study. Block-randomization was performed for study subjects with < 200 versus 200–350 cells/μL at screening. The MVC dose was 150–600 mg twice daily, depending on interactions with concurrent medication, as specified in the package insert. In case of virological failure (defined as two consecutive plasma HIV-1 RNA measurements of 50 copies/mL or higher), participants had to discontinue study medication. Subjects were seen for screening, and at baseline, weeks 2, 4, 8, 12, 24, 36 and 48. At all visits, patients were questioned for side effects and other complaints, physical examination (if indicated) was conducted, and EDTA- and heparin-plasma was drawn. Adherence to study drug was assessed at every visit by self-report, and additionally at week 4, 12, 24, 36 and 48 by pill count.

### Virologic analyses

Plasma HIV-RNA was measured in the participating sites using standard commercial assays with a lower limit of detection of 20–40 copies/mL. For genotypic prediction of HIV-1 coreceptor tropism, viral DNA was isolated from 5×106 PBMCs. The V3 region of the viral envelope was amplified as previously reported [24]. In brief; V3 region was amplified with primers 6206V3F 5’-AGAGCAGAAGACAGTGGCAATGAGAGTGA-3’, 7785R 5’-AGTGCTTCCTGCTGCTCCYAAGAACCC-‘3 (Titan One Tube RT-PCR kit, Roche; no RT reaction performed). The nested-PCR was performed with primers 6658F 5’-TGGGATCAAAGCCTAAAGCCATGTG-‘3, 7371R 5’-GAAAATTCCCCTCCACAATT-‘3 (Expand High-Fidelity PCR-System, Roche). Sequenced using primers 6957F 5’-GTACAATGTACACATGGAAT-‘3 and 7371R or V3-4 5’-ACAGTACAATGTACACATGGAATTA-3’ and V3-3 5’-AATTCCCCTCCACAATTAAAASTGTG-3’ (Big dye Terminator Cycle seq kit v3,1, Applied Biosystems). Viral co-receptor tropism was predicted in triplicate on samples of week 48 using ‘geno2pheno [co-receptor] algorithm’ (http://www.geno2pheno.org/). False positive rate (FPR) cutoff-values for CCR5 or CXCR4 prediction were used according to European guidelines [25] (10% for plasma HIV-RNA, 20% when only one sequence could be generated in samples with plasma HIV-RNA < 1000 cp/mL; 10% for plasma HIV-DNA, 20% when only one sequence could be generated). PCR amplification and sequencing was performed in triplicate. When co-receptor tropism of the proviral DNA population was predicted to be CXCR4-tropic, baseline samples were analyzed to investigate whether a switch in co-receptor tropism from CCR5 to CXCR4 had taken place during the study treatment period.

### Immunologic analyses

Absolute CD4^+^ T-cell counts were assessed by flow cytometry at the local-site laboratory at each visit. Peripheral blood mononuclear cells (PBMCs) were isolated from whole blood via density gradient centrifugation, cryopreserved and stored. Total cell numbers and subsets (i.e. naive (CD27^+^ CD45RO^-^), memory (CD45RO^+^) and effector (CD27^-^CD45RO^-^)), and the expression of markers for activation (CD38^+^/HLA-DR^+^), proliferation (Ki-67^+^), apoptosis (annexin-V^+^) and HIV-1 coreceptors (CCR5^+^ and CXCR4^+^) were determined for CD3^+^CD4^+^ and CD3^+^CD8^+^ T cells. We measured the expression of CD31^+^ within the naive T cell population as an indication of thymic T-cell production.

Levels of soluble CD14 and soluble CD163 in plasma were assessed as a measure of monocyte activation and the level of soluble IL2 receptor (sIL2R) as a measure of T cell activation. Plasma levels of interferon-γ induced protein 10 (IP10) were determined as a measure of (residual virally induced) chronic inflammation.

T-cell subsets and CD31^+^ analysis were in principal performed on fresh PBMCs obtained at every visit of all participants. The remaining T-cell markers were assessed in an in depth T-cell analysis performed in a subset of patients (selected on basis of sample availability) on thawed material by flow cytometry as described previously [26]. Frozen PBMCs from visits at week 0, 2, 4, 24 and 48 were used. In case a sample was not available of the given time points, a sample of the closest available time point was chosen. Flow cytometry was performed using a FACS LSR II (BD Biosciences) and FACS Diva software (BD Biosciences). Lymphocytes were gated based on forward and side scatter and subsets were identified based on the expression of a combination of molecules (S1 Table, S1 and S2 Figs). Plasma markers were assessed on heparin plasma (stored at -80°C, once or twice thawed). The concentration of soluble CD14 and soluble CD163 was determined using a commercial ELISA kits (Gen-Probe Diaclone SAS and Trillium Diagnostics LLC). The CCR5-ligands CCL5 (RANTES (regulated on activation, normal T cell expressed and secreted)), MIP-1alpha and MIP-1beta were measured in a subset of patients (selection based on sample availability) at baseline, week 4 and week 48 using a Luminex assay. Samples were diluted 100 (CCL5) or 10 (MIP-1 alpha and beta) fold to prevent prozone effects in the assay [27].

### Sample size calculation

We determined the number of individuals needed in each arm to detect a 30% difference in increase in CD4^+^ T-cell count (primary outcome) with a standard-deviation of 30 and 40% (placebo and treatment arm respectively) and a power of 0.90 (n = 62). Two patient groups were defined based on the inclusion criteria for CD4^+^ T cell count. We aimed at including sufficient patients in both groups to analyze these groups separately and anticipating a potential loss to follow-up and early treatment discontinuations of 10%, we therefore planned to enroll 130 patients.

### Statistical analyses

Primary outcome was the change in absolute CD4^+^ T-cell count. An intention-to-treat (ITT) analysis was performed, in case of premature discontinuation of the study CD4^+^ T-cell counts after discontinuation until week 48 were included.

Continuous variables were compared using a Student’s t-test or a Mann-Whitney test (in case of non-normal distribution or a sample size lower than 30), while for categorical variables a Chi-square or Fisher’s exact test was used. Differences were considered statistically significant at p < 0.05.

Changes in T-cell numbers, T-cell markers, soluble markers and CCR5-ligands were studied using data of all analyzed time-points using linear mixed effects models including fixed and random effects on both intercept and slopes with unstructured correlation matrix. Trends in the evolution of markers were fitted using one or two slopes depending on the best fit (defined by Akaike criteria, the lower the better). The time taken for the slope to change was determined for all patients by likelihood profile. To achieve normality and homoscedasticity of measurement error distributions, the fourth-root of markers was used instead of the natural markers when necessary. Statistical analyses were performed with SAS software (version 9.2, SAS institute Inc.) and SPSS statistics 20. ‘p_m_’ and ‘p_p_’ denote p-values of change over time (start to week 48) within the maraviroc and placebo arm respectively. ‘p_a_’ denotes the p-value of the comparison between the arms.

## Results

### Study population and safety

**Fig 1:**
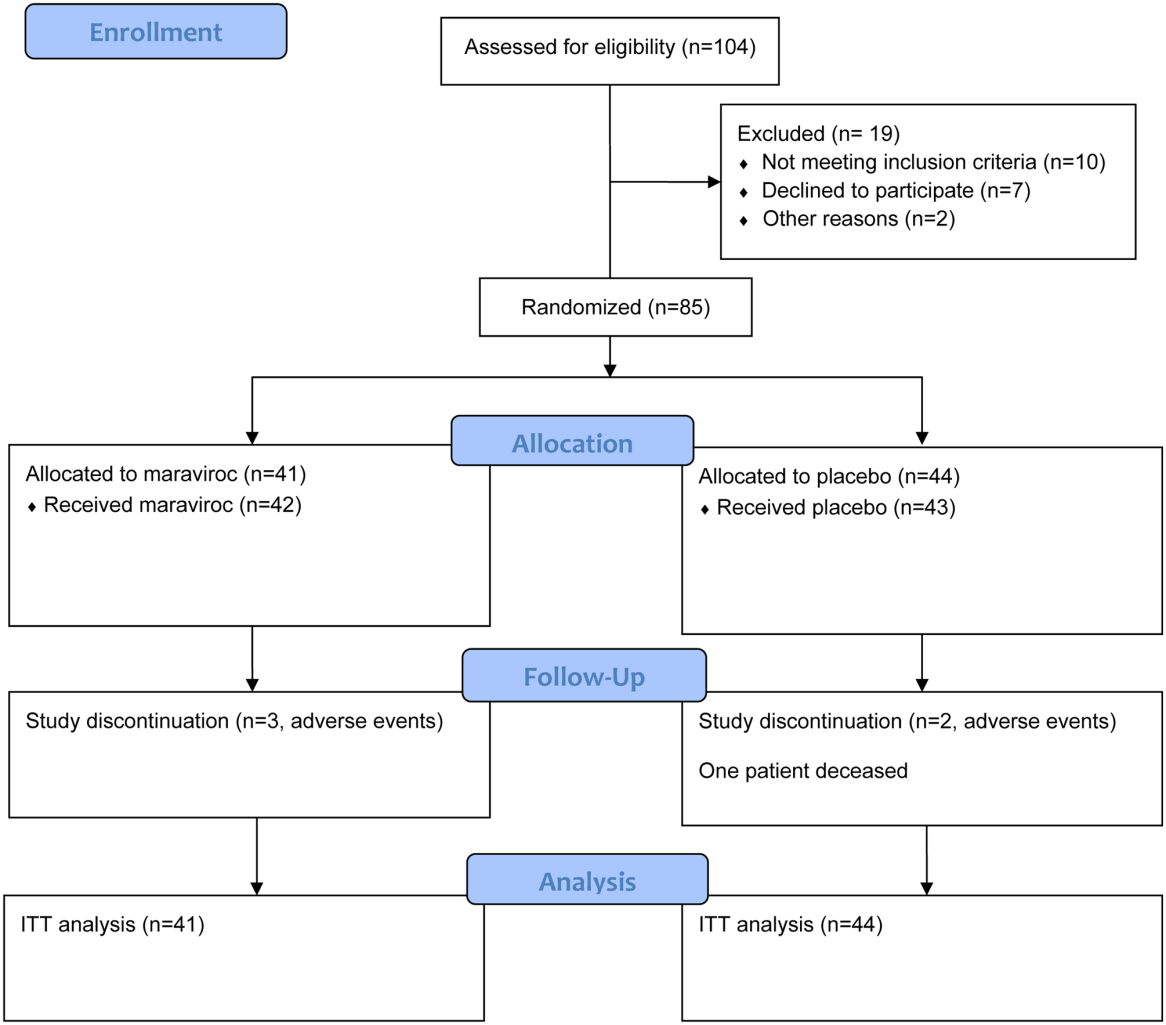
CONSORT 2010 flow diagram of the study population. One hundred and four patients were screened, of whom 85 were included in the study. One patient was mistakenly treated with MVC instead of placebo during the entire study and an intention-to-treat analysis was performed. One participant deceased (in the placebo arm) whereas 5 participants prematurely discontinued the study (at week 2, 13, 18, 29 and 42); 79 study participants finished the complete study protocol. Data of all participants were included in the analysis of the primary endpoint.

Between February 2009 and February 2011 one hundred and four patients were screened for eligibility. Slow enrolment restricted inclusion to a final of 85 patients. One patient was mistakenly treated with MVC instead of placebo during the entire study and an intention-to-treat analysis was performed for the primary endpoint (Fig 1).

The study medication was well tolerated. During the study 16 serious adverse events were registered in 12 study participants (7 in the placebo and 9 in the MVC arm (p_a_ = 0.55)). In two cases the study medication could initially not be ruled out as a causative factor. However, the gastro-intestinal side effects that led to study discontinuation in one participant were caused by *Giardia Lamblia* infection, and the study participant recovered completely after antiparasitic treatment. In the other participant, plasma gamma-glutamyl transpeptidase (γGT), which was already elevated at the start of the study (800 U/L, >10 times upper limit of normal), temporarily increased to 1607 U/L. This study participant was known to have a large alcohol intake, liver biopsy showed signs of steatotic hepatitis. It was therefore decided to continue his study medication, and after he decreased his alcohol intake the γGT returned to pre-study levels. One patient deceased (in the placebo arm) by natural death of unknown cause as classified by the coroner. Five patients discontinued the study medication because of side effects (n = 3), no effect on CD4^+^ T-cell count (n = 1) or other reasons (n = 1). Seventy-nine patients completed the full study period.

Baseline characteristics did not differ significantly between the study arms, except for baseline effector CD8^+^ T-cell counts, which were significantly higher in the maraviroc arm. HIV co-receptor tropism prediction was not significantly different between the study arms (Table 1). None of the study participants experienced virological failure and no changes in HIV co-receptor tropism were observed during the study period.

**Table 1:** Clinical, immunological and virological baseline characteristics of the study participants.

	Total	Placebo	Maraviroc	P-value
N	85	44	41	
Age (years)	49 (43–57)	51 (43–59)	48 (41–54)	0.07
Male sex, n (%)	80 (94)	43 (98)	37 (90)	0.19
Years since start cART	5.1 (3.2–9.9)	5.0 (3.2–8.9)	6.0 (3.0–12.4)	0.45
Years since last detectable VL	3.4 (2.2–5.9)	4.0 (2.3–6.8)	3.3 (2.1–5.5)	0.53
Previous CDC-C events	48 (56.5)	26 (60.5)	22 (52.4)	0.51
AZT containing cART, n(%)	4 (4.7)	3 (6.8)	1 (2.4)	0.34
HCV co-infection, n(%)^a^	6 (7.1)	3 (6.8)	3 (7.3)	0.66
Nadir CD4+ T cells	40 (10–86)	30 (10–80)	47 (10–90)	0.55
CD4+ T cells	237 (180–286)	225 (178–290)	240 (180–286)	0.59
Plasma HIV-RNA^b^	< 50	< 50	< 50	
Naive CD4+ T cells	49 (28–73)	47 (21–73)	50 (33–73)	0.47
Memory CD4+ T cells	169 (133–211)	168 (126–217)	172 (134–208)	0.92
Effector CD4+ T cells	3 (2–13)	3 (1–16)	6 (2–12)	0.40
CD8^+^ T cells	837 (597–1210)	719 (503–1107)	970 (719–1212)	0.08
Naive CD8^+^ T cells	151 (95–213)	146 (62–262)	154 (116–189)	0.52
Memory CD8^+^ T cells	424 (221–653)	352 (210–584)	438 (276–655)	0.67
Effector CD8^+^ T cells	179 (75–385)	158 (54–259)	289 (95–507)	0.03
R5-tropism, n(%)^c^	28 (49)	15 (48)	13 (50)	0.90
X4-tropism, n(%)^c^	29 (51)	16 (52)	13 (50)	0.90

Values are given as median (interquartile range). T-cell counts are given as cells/μL and plasma HIV-RNA as copies/mL.

^a^Of 5 patients HCV status was unknown (4 placebo, 1 MVC).

^b^Plasma HIV-RNA was measured at screening visit (and at week 4, 12, 24, 36, 48).

^c^Samples of 63 patients were used for HIV co-receptor tropism prediction, which was successful in 57 patients. For T-cell subsets, data from on average 91.2% of all time points were available.

Abbreviations: cART = combination antiretroviral therapy; VL = plasma viral load; R5-tropism: CCR5-tropic virus population; X4-tropism: CXCR4-tropic virus population.

### Changes in CD4^+^and CD8^+^ T-cell counts and subsets

**Fig 2:**
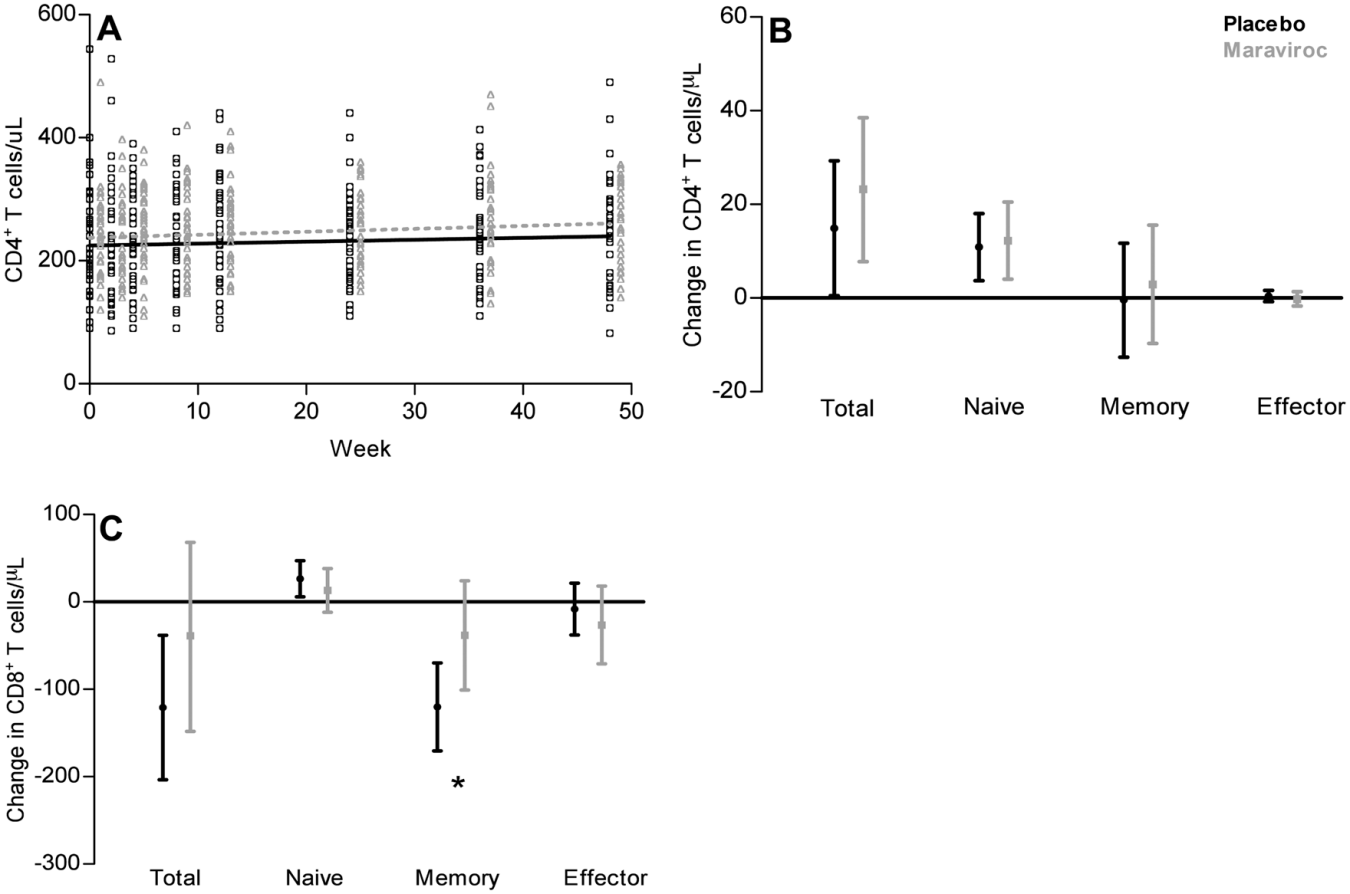
A. Change in CD4^+^ T-cell counts during 48 weeks of intensification of cART with maraviroc or placebo. The open dots (placebo arm) and triangles (MVC arm) represent individual CD4^+^ T-cell measurements. The lines (black placebo, grey MVC-arm) represent the dynamics determined by linear mixed effects models. B-C. Mean change (95% Confidence Intervals) based on linear mixed effects models in total, naive, memory and effector CD4^+^ (A) and CD8^+^ (B) T-cell counts in 48 weeks of MVC treatment intensification. Changes in the placebo arm are in black and in the MVC arm in grey. Asterisks indicate significant differences between the arms (p<0.05).

Linear mixed effect model analysis showed a significant increase of 15.3 CD4^+^ T cells/μL (95% CI [1.0, 29.5]) in the placebo arm versus 22.9 CD4^+^ T cells/μL (95% CI [7.4, 38.5]) in the MVC arm over the treatment period. These increases were not significantly different between both arms (p_a_ = 0.51; Fig 2a). Naive CD4^+^ T-cell counts (Fig 2b) increased similarly in the placebo and the MVC arm (p_a_ = 0.98): +10.9 cells/μL (95% CI [3.7, 18.0]) in the placebo arm versus +12.2 cells/μL (95% CI [4.0, 20.5]) in the MVC arm. Neither memory nor effector CD4^+^ T-cell counts changed significantly in either of the two arms during the study period. No significant difference in CD4^+^ T cell response was observed between study participants harbouring R5- versus X4 tropic viral populations.

For CD8^+^ T-cells (Fig 2c), a significant decrease of 120.8 cells/μL (95% CI [-203.6, -38.1]) was observed in the placebo arm, whereas total CD8^+^ T-cell counts remained constant in the MVC arm (p_m_ = 0.50). Neither naive nor effector CD8^+^ T-cell counts changed significantly in either of the arms. However, memory CD8^+^ T-cell counts significantly decreased in the placebo arm (-120.2 cells/μL, 95% CI [-170.4, -70.0]), while in the MVC arm this subset did not change (p_m_ = 0.33).

### Effects on T-cell characteristics

To investigate the effects of maraviroc intensification on cART on T-cell production, proliferation, activation and death, we performed an extended analysis in a subset of 67 patients which as a group was comparable to the total study population. At baseline, the expression of none of these markers differed significantly between the treatment arms (Table 2).

**Table 2:** Baseline T-cell characteristics and marker levels.

	Total	Placebo	Maraviroc	P-value^a^
N	67	34	33	
CD38^+^ HLA-DR^+^ CD4^+^ T cells	3.1 (2.3–4.4)	3.1 (2.2–4.0)	3.1 (2.5–4.5)	0.78
CD38^+^ HLA-DR^+^ CD8^+^ T cells	5.0 (2.8–8.1)	5.8 (2.8–10.2)	4.9 (3.0–7.1)	0.55
Ki67^+^ CD4^+^ T cells	3.3 (2.0–4.3)	3.4 (2.2–4.3)	3.2 (1.9–4.2)	0.52
Ki67^+^ CD8^+^ T cells	1.0 (0.7–1.6)	1.0 (0.7–1.9)	1.2 (0.8–1.6)	0.69
CD31^+^ naive CD4^+^ T cells	56.3 (41.8–66.6)	57.7 (44.9–64.6)	54.1 (40.7–66.6)	0.81
Annexin-V^+^ CD4^+^ T cells	20.4 (14.2–27.4)	19.0 (13.1–26.1)	20.7 (15.4–28.2)	0.39
Annexin-V^+^ CD8^+^ T cells	34.5 (20.3–47.6)	33.7 (14.3–46.1)	35.7 (26.7–51.3)	0.27
CCR5^+^ CD4^+^ T cells	4.7 (1.8–9.1)	5.0 (2.1–7.2)	4.3 (1.8–12.0)	0.69
CCR5^+^ CD8^+^ T cells	12.9 (7.0–21.9)	14.1 (7.4–21.9)	11.5 (6.0–15.5)	0.41
CXCR4^+^ CD4^+^ T cells	42.2 (27.3–62.1)	42.2 (28.0–65.0)	45.7 (24.9–61.6)	0.89
CXCR4^+^ CD8^+^ T cells	34.5 (16.1–62.3)	32.6 (17.2–58.4)	36.3 (14.1–63.5)	0.83
sCD14 (μg/L)^b^	5.8 (4.9–10.0)	7.9 (5.7–9.5)	7.2 (5.8–10.3)	0.98
sCD163 (pg/mL)^c^	1.6 (1.1–2.1)	1.5 (1.0–2.1)	1.7 (1.2–2.7)	0.19
MIP-1α (pg/mL)^d^	23.3 (14.4–38.1)	22.3 (12.4–39.8)	23.3 (14.4–42.2)	0.93
MIP-1β (pg/mL)^e^	78.5 (41.0–122.6)	61.4 (26.4–115.0)	88.4 (51.4–148.5)	0.23
CCL5 (ng/mL)^e^	257.0 (176.9–371.3)	227.3 (169.7–319.5)	268.5 (190.9–435.1)	0.23

Expression of T-cell markers is given as a percentage (%). Values are given as median (interquartile range).

^a^P-value of MVC compared to placebo arm.

^b^Measured in 80 (41 placebo and 39 MVC),

^c^49 (25 placebo and 24 MVC),

^d^44 (22 placebo, 22 MVC) and

^e^46 patients (24 placebo and 22 MVC).

**Fig 3:**
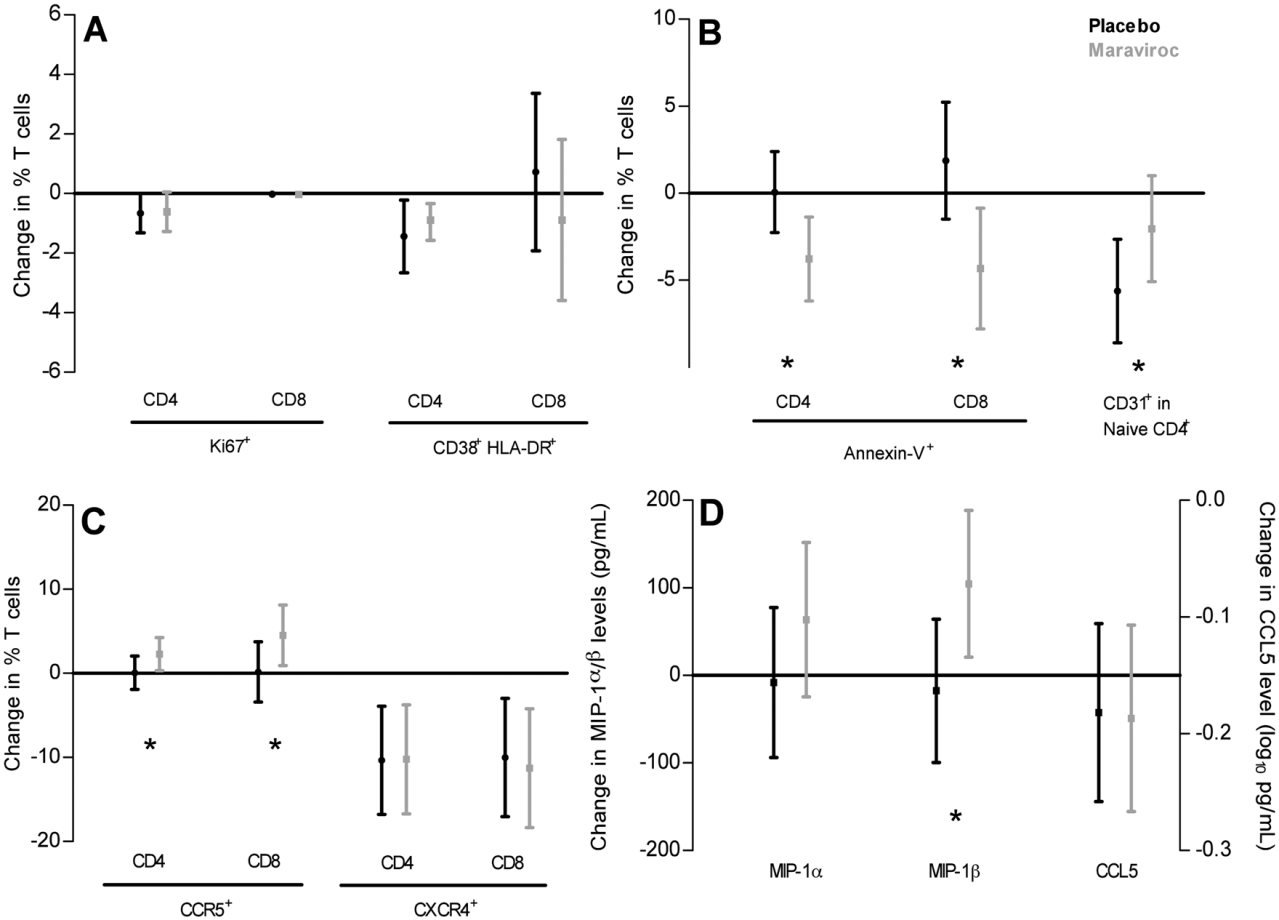
Mean change (95% Confidence Intervals) based on linear mixed effects models in percentages of T cells expressing markers for (A) proliferation (%Ki67^+^), activation (%CD38^+^ HLA-DR^+^), (B) apoptosis (Annexin-V^+^), thymus proximity (%CD31^+^ within the naive CD4^+^ T-cell population) and (C) the percentage of CCR5^+^ and CXCR4^+^ T cells in 48 weeks of maraviroc treatment intensification. In 59 out of 67 patients (88%) data from 5 time points were available, in the remaining 8 patients 4 time points were available. Mean change (95% Confidence Intervals) based on linear mixed effects models of plasma levels of the CCR5-ligands MIP-1α, MIP-1β and CCL5 (log_10_ transformation) during the study period (D). Changes in the placebo arm are in black and in the MVC arm in grey. Asterisk indicates a significant differences between the arms (p <0.05).

The percentage of CD38^+^ HLA-DR^+^ CD4^+^ T cells decreased significantly by -1.4% (95% CI [-2.7, -0.2]) in the placebo arm, which was comparable (p_a_ = 0.57) to the -0.3% (95% CI [-1.6, -0.9]) decrease observed in the MVC arm (Fig 3a). Within the CD8^+^ T-cell pool, the percentage of CD38^+^ HLA-DR^+^ cells remained constant in both arms (p_p_ = 0.59 and p_m_ = 0.52). At week 24, no significant difference in T-cell activation levels between the arms was found either. The percentage of Ki67^+^ CD4^+^ and Ki67^+^ CD8^+^ T cells did not change significantly in either one of the arms (Fig 3a).

With respect to apoptosis, both for CD4^+^ and CD8^+^ T cells the percentage of Annexin-V^+^ cells significantly decreased in the MVC arm (CD4^+^ -3.8%, 95% CI [-6.2, -1.3], CD8^+^

-4.3% (95% CI [-7.8, -0.9]), while it remained constant in the placebo arm (CD4^+^ p_p_ = 0.96, CD8^+^ p_p_ = 0.27, Fig 3b).

Since CD31^+^ naive T cells are thought to be more proximal to the thymus than their CD31^-^ counterparts [28], we also followed the change in the percentage of CD31^+^ naive CD4^+^ T cells during treatment intensification. We observed a significant decrease in the placebo arm (p_p_ = 0.0002) by 5.6% (95% CI [-8.6, -2.6]) while it remained constant (p_m_ = 0.19) in the MVC arm (p_a_ = 0.0002; Fig 3b).

In both arms the plasma concentration of soluble CD14 decreased significantly: -1.5 μg/L (95% CI [-2.0, -0.9]) in the placebo arm versus -0.7 μg/L (95% CI [-1.3, -0.2]) in the MVC arm (p_a_ = 0.06). Although the plasma concentration of soluble CD163 increased significantly in the MVC arm during the study period (0.08 95% CI [0.013, 0.15]; p_m_ = 0.02), this was not significantly different as compared to the placebo arm (0.04 95% CI [-0.03, 0.11]; p_p_ = 0.23, p_a_ = 0.06). No difference in changes of immune activation markers sIL2R and IP10 were observed in and between the study arms.

In summary, MVC intensification i) prevented the decrease of the percentage of CD31^+^ naive CD4^+^ T cells during treatment, and ii) decreased the percentage of apoptotic Annexin-V^+^ CD4^+^ and CD8^+^ T cells.

### CCR5^+^ and CXCR4^+^ expression on T cells and CCR5 ligand plasma levels

The presence of maraviroc did not alter the expression of CXCR4 as the percentage of CXCR4^+^ expressing CD4^+^ (p_a_ = 0.98) and CD8^+^ T cells (p_a_ = 0.80; Fig 3c) declined similarly in both arms. For CCR5 expression on CD4^+^ and CD8^+^ T cells a significant increase was only observed in the MVC arm (2.3% (95% CI [0.3, 4.2]) and 4.5% (95% CI [0.9, 8.1]) respectively). The increase for CD4^+^ T cells was mainly observed during the first two weeks of treatment (0.93% per week, 95%CI [0.24, 1.63]; p_m_ = 0.009).

The levels of the CCR5 ligands CCL5, MIP-1α and MIP-1β were assessed in a sub-analysis in a representative subgroup of 46 study subjects (24 placebo and 22 MVC). MIP-1β levels significantly increased in the MVC arm (p_m_ = 0.02), whereas no significant change was observed in the placebo arm (p_p_ = 0.66; 104.6 (95% CI [20.9, 188.3]) versus -17.7 (95% CI [-99.1, 64.1]) pg/ml; p_a_ = 0.04). MIP-1α levels increased in the MVC arm as well, however this was not significant (63.7 95% CI [-24.6, 152.0]; p_m_ = 0.15). No differences were found between the arms for MIP-1α and CCL5 (Fig 3d).

## Discussion

In this randomized, placebo-controlled trial, we investigated the effect of MVC intensification on CD4^+^ T-cell recovery in patients with a suboptimal immunological response despite viral suppression. No significant effect on CD4^+^ T-cell gain during 48 weeks of MVC intensification of cART was observed. MVC intensification did, however, influence the immune system: it increased CCR5 expression on both CD4^+^ and CD8^+^ T cell and decreased T-cell apoptosis levels. Moreover it reduced the loss of CD8^+^ memory T cells and counteracted the decrease in CD31^+^ naive CD4^+^ T cells. Possible clinical implications of these findings are not yet clear and might be directly related to modulation of ligand induced CCR5 signaling rather than being result of indirect effects on HIV replication and/or production.

Besides the fact that the CCR5 receptor is a co-receptor for HIV-1 [29,30] this receptor has also been shown to directly affect T cells. Binding of chemokines to CCR5 stimulates T-cell migration, co-stimulates T-cell activation [10,11] and modulates apoptosis [31]. Potential immunomodulatory effects of MVC could thus act via further suppression of residual low level viremia (replication and/or production), via direct blocking of the normal physiological functions of the CCR5 receptor on T cells or a synergistic combination of both mechanisms. To our knowledge six other MVC intensification studies in immunological non-responders have been published in peer-reviewed journals [17,19–22]of which only one was placebo-controlled [20]. In this 24 week placebo-controlled trial 45 patients with a median baseline CD4^+^ T-cell count of ≈200 CD4 cells/μL were included. In agreement with our results they observed a modest increase in CD4^+^ T cells in both arms (placebo 17 (95% CI [17–127;] p = 0.008), versus 17 (95% CI, [17 –28]; p = 0.004) cells/μL in the maraviroc arm) and an increase of CCR5 expression in the maraviroc arm. CD4^+^ and CD8^+^ T-cell activation (CD38^+^HLA-DR^+^) was determined in peripheral blood as well as gut mucosa and increased especially in the gut mucosa during maraviroc intensification. It was suggested that blocking of the CCR5 receptor led to an increased level of chemokines (as measured by an increase in MIP-1β in the MVC arm) as a result of decreased internalization of the CCR5 receptor-ligand complexes, resulting in an increase in T-cell activation via other ligand-dependent pathways. In agreement with this data we found an increase in the frequency of CCR5^+^ T cells and MIP-1β levels in the MVC arm, as well as a non-significant increase of MIP-1α. However, we did not observe a change in T-cell activation levels during MVC intensification as compared to placebo, both at week 24 and week 48. Although the study population included in our study seems comparable in terms of CD4^+^ T-cell count, age and sex, the average time on cART at baseline was 2–3 fold longer and T-cell activation levels were 2–3 fold lower. Longitudinal analysis has shown that T-cell activation levels decrease during cART [32–34] and continue to do so up to 5 years after initiation of cART [unpublished results]. The difference in time on cART between the studies might thus explain the difference in baseline T-cell activation. Why T-cell activation is differently affected by MVC intensification in these different baseline situations remains unclear.

In a French (MARIMUNO-ANRS 145 Study [21]) and U.S. (ACTG A5256) [19] open label single arm studies modest increases in CD4^+^ T-cell counts were found during MVC intensification. However since we found a comparable increase in the placebo arm, this does not seem to be the result of MVC intensification but rather reflects the normal average CD4^+^ T-cell increase over time. Moreover, we could not confirm the effect of MVC intensification on the reduction of T-cell activation or markers of monocyte activation as observed in previous studies of shorter duration (24 weeks) [19,21,35]. In our study CD38^+^ HLA-DR^+^ CD4^+^ T cells and soluble CD14 levels in the MVC arm decreased, however this was not significantly different compared to the placebo arm. Thus, emphasizing the need for placebo-controlled studies.

The increased expression of CCR5 in the CD4^+^ and CD8^+^ T cell populations and the increase in MIP-1β levels during treatment with a CCR5 receptor antagonist as observed in our and other studies [20,36], are in line with the MVC-induced CCR5 up-regulation on T cells in vitro [37] and might be the consequence of disruption of the CCL5-CCR5 interaction [36]. This interaction has been shown to result in internalisation of the CCR5 receptor and to inversely correlate with CCR5 expression on the T cell surface. Of note, MIP-1α levels increased as well during the study, although this was not significant.

High levels of spontaneous apoptosis in acute HIV infection is closely associated with increases in CCR5 and decreases in Bcl-2 and interleukin (IL)–7 receptor levels [38]. Studies by Algeciras-Schimnich and Murooka have shown that cross-linking of CCR5 can modulate apoptosis and suggest that CCR5 might do more than marking (recently activated) apoptosis prone cells [31,39]. We observed a significant decrease in the level of expression of the apoptosis marker Annexin-V on CD4^+^ and CD8^+^ T cells in the MVC arm only, and a reduction in the decrease of CD8^+^ memory T cells. In the single-arm open-label ACTG A5256 trial [19] an increase in the percentage of CD4^+^ and CD8^+^ T cells expressing the anti-apoptotic marker Bcl-2 and a decrease in the percentage of CD4^+^ and CD8^+^ T cells expressing the apoptosis marker caspase-3 was observed, consistent with reduced levels of apoptosis in T cells. If CCR5 indeed is important in the regulation of T-cell apoptosis, then apoptosis levels might decrease when CCR5 activation is blocked by MVC. The relatively high levels of CCR5 on memory CD8^+^ T cells and the observed reduction in the decrease of memory CD8^+^ T-cell numbers in the MVC arm is in line with such a mechanism.

Beside a reduction of apoptosis, blocking of CCR5 with antagonists has been shown to reduce chemotaxis *in vitro* and reduce T-cell trafficking *in vivo*. Reshef et al. have shown a reduced risk of visceral acute graft-versus-host disease due to inhibition of lymphocyte trafficking in recipients of allogeneic hematopoietic stem-cell transplantation by addition of MVC to the treatment regimen [12]. Similarly treatment with the CCR5-blocker SCH532706 reduced T-cell and plasmacytoid dendritic cell trafficking in HIV-infected patients [40]. In our study decreased trafficking out of the blood could have altered CCR5^+^ T-cell numbers in blood and caused the slower the decline of memory CD8+ T-cells in the MVC arm.

Ahuja et al. studied the effect of variations in CCL3L1 gene dose and CCR5 genotype on immune reconstitution after initiation of cART [13]. Although CD4^+^ T-cell count increased regardless of genotype during the first two years of cART, thereafter patients with a favorable genotype showed a significantly more durable CD4^+^ T-cell recovery. Therefore, it might be possible that the duration of this trial is too short to find a significant effect of MVC intensification of cART on CD4^+^ T-cell recovery, especially in the included study population (patients with a slow immunological response on cART). This might explain our observation that MVC intensification reduces T-cell apoptosis, whereas we did not find an effect on CD4^+^ T-cell recovery.

In our study, none of the study participants experienced virological failure and since other studies did not find an effect of maraviroc intensification on residual plasma HIV-RNA [18,20,21], we think that the observed effects on T-cell apoptosis are the consequence of a direct blockade of CCR5 signalling on T cells and not an indirect effect of changes in plasma HIV-RNA (levels) by MVC intensification. In line with these observations, a sub analysis based on co-receptor tropism showed no effect of baseline co-receptor tropism on CD4^+^ T-cell restoration.

This study has its limitations. The number of patients that has been included was lower than planned and small but significant improvements in CD4^+^ T-cell gain by MVC intensification may not have been detected. Since CD4^+^ T-cell gain can be slow but persistent in patients with very low CD4^+^ T-cell counts at start cART, longer duration of the study might have augmented MVCs effects on CD4^+^ T-cell gain and revealed these smaller changes. Of note, all other maraviroc intensification studies have a shorter follow-up. The low numbers of patients might also have influenced the outcomes in the immunological sub-study and may underlie the observed differences of the effect of MVC intensification on the expression of T cell activation markers between studies of different laboratories.

In conclusion, the results of this largest, placebo-controlled MVC intensification trial to date do not support MVC intensification of cART in patients with a suboptimal immunological response in order to enhance restoration of CD4^+^ T-cell counts. However, since we did observe an increase in CCR5^+^ T cells and MIP-1β levels, as well as a decrease in T-cell apoptosis levels in the MVC arm, further (placebo-controlled) studies are needed to investigate whether these findings have any clinical consequences.

## Supporting Information

S1 CONSORT Checklist CONSORT checklist. (DOC)

S1 Fig Gating strategy for measuring T cell subsets and characteristics by flow cytometry. *Upper panel*: general gating strategy for CD8^+^ and CD4^+^ T cells. *Panel 1*: Gating strategy of CD4^+^ and CD8^+^ T cell subsets (naive [CD27^+^CD45RO^-^], memory [CD45RO^+^] and effector [CD27^-^CD45RO^-^]). Gating strategy of activated (CD38^+^, HLA-DR^+^) CD4^+^ and CD8^+^ T cells. *Panel 2*: Gating strategy for CD31^+^ naive CD4^+^ T cells as an indication of thymic T-cell production.
(TIF)

S2 Fig Gating strategy for measuring T cell subsets and characteristics by flow cytometry. *Panel 3*: Gating strategy for CCR5^+^ and CXCR4^+^ CD8^+^ and CD4^+^ T cells. *Panel 4*: Gating strategy for apoptotic (Annexin-V^+^, 7AAD^-^) CD4^+^ and CD8^+^ T cells. *Panel 5*: Gating strategy for proliferating (Ki67^+^) CD4+ and CD8+ T cells.
(TIF)

S1 Protocol Study protocol. (PDF)

S1 Table Staining panels and antibodies used. (DOCX)
