# Supplementary figures and images for: Maraviroc Intensification of cART in Patients with Suboptimal Immunological Recovery: A 48-Week, Placebo-Controlled Randomized Trial
Source: PLoS One. 2015 Jul 24;10(7):e0132430. doi: 10.1371/journal.pone.0132430 (PMC4514679; doi:10.1371/journal.pone.0132430)

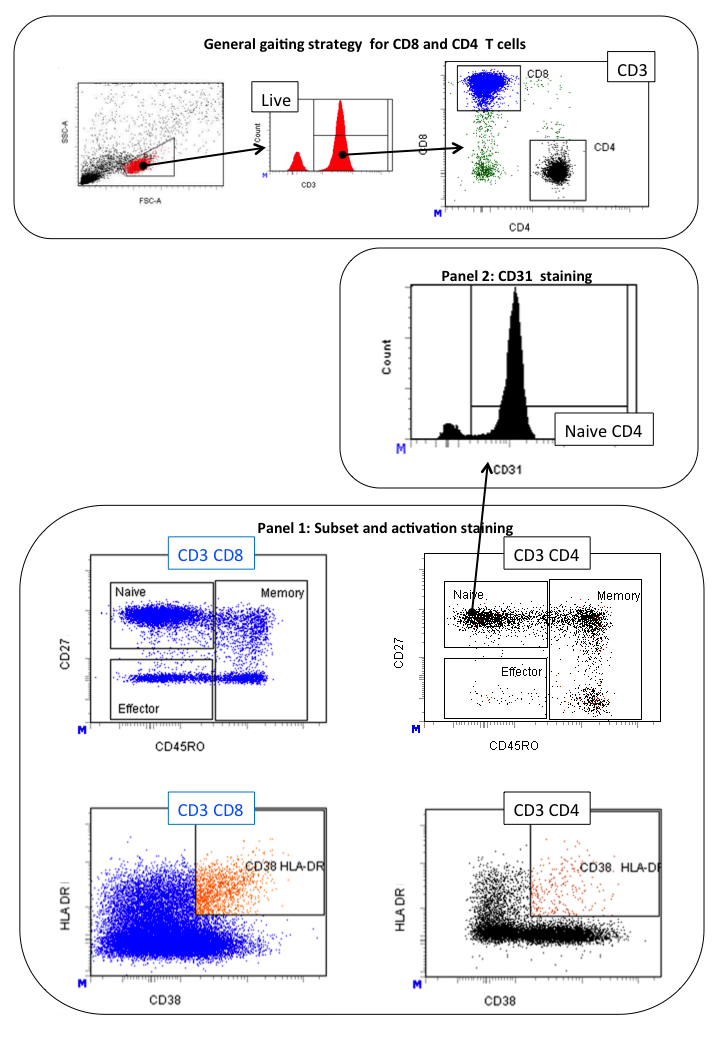

Supplement: S1 Fig — Upper panel: general gating strategy for CD8+ and CD4+ T cells. Panel 1: Gating strategy of CD4+ and CD8+ T cell subsets (naive [CD27+CD45RO-], memory [CD45RO+] and effector [CD27-CD45RO-]). Gating strategy of activated (CD38+, HLA-DR+) CD4+ and CD8+ T cells. Panel 2: Gating strategy for CD31+ naive CD4+ T cells as an indication of thymic T-cell production. (TIF) [file pone.0132430.s002.tif]

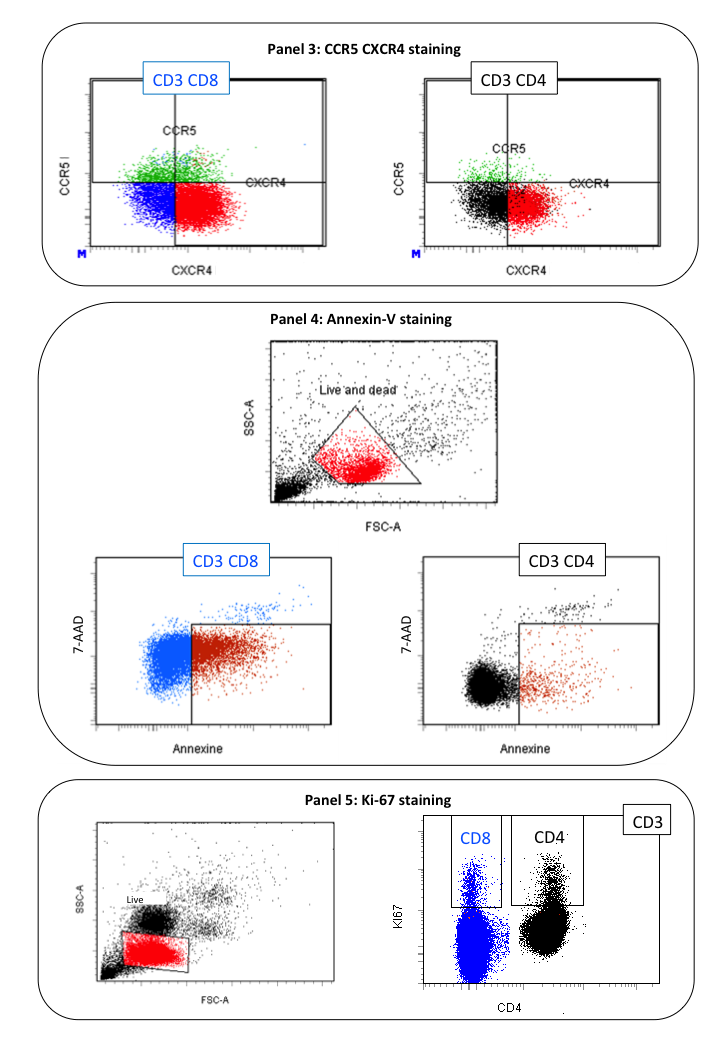

Supplement: S2 Fig — Panel 3: Gating strategy for CCR5+ and CXCR4+ CD8+ and CD4+ T cells. Panel 4: Gating strategy for apoptotic (Annexin-V+, 7AAD-) CD4+ and CD8+ T cells. Panel 5: Gating strategy for proliferating (Ki67+) CD4+ and CD8+ T cells. (TIF) [file pone.0132430.s003.tif]
