# Supplementary material for: Maraviroc Intensification of cART in Patients with Suboptimal Immunological Recovery: A 48-Week, Placebo-Controlled Randomized Trial
Source: PLoS One. 2015 Jul 24;10(7):e0132430. doi: 10.1371/journal.pone.0132430 (PMC4514679; doi:10.1371/journal.pone.0132430)
Supplement: S1 Protocol — (PDF) [file pone.0132430.s004.pdf]

Maraviroc Immune Recovery Study (MIRS): A multicenter, randomized, placebo-controlled, exploratory mechanistic study into the role of maraviroc on immune recovery

(May 25, 2009)

PROTOCOL TITLE 'Maraviroc Immune Recovery Study: A multicenter, randomized, placebo-controlled, exploratory mechanistic study into the role of maraviroc on immune recovery'

Protocol ID	MIRS2008
Short title	MIRS
Version	May 25, 2009
Date	25-05-2009
Coordinating investigator/project leader	S.F.L. van Lelyveld<br>Interne Geneeskunde & Infectieziekten<br>UMC Utrecht<br>Huispostnummer F.02.126<br>Heidelberglaan 100<br>3584 CX Utrecht
Principal investigators	Prof. Dr. I.M. Hoepelman<br>Interne Geneeskunde & Infectieziekten<br>UMC Utrecht<br>Huispostnummer F.02.126<br>Heidelberglaan 100<br>3584 CX Utrecht
Sponsor	UMC Utrecht
Independent physician(s)	Dr. E.J.G. Peters<br>Internist-infectioloog<br>UMC Utrecht<br>Huispostnummer F.02.126<br>Heidelberglaan 100<br>3584 CX Utrecht

Laboratory sites	Immunology<br><br>Mw. Dr. K. Tesselaar, immunologist<br><br>UMC Utrecht, locatie WKZ<br><br>Lundlaan 6<br><br>3584 EA Utrecht<br><br><br>Medical Microbiology, department of virology<br><br>Mw. Dr. M. Nijhuis, virologist<br><br>Mw. Dr. A.M.J. Wensing, clinical virologist<br><br>UMC Utrecht, locatie AZU<br><br>Heidelberglaan 100<br><br>3584 CX Utrecht
Pharmacy	Mw. S. El Yandouzi, pharmacist<br><br>Department of clinical trial support<br><br>Hoofddijk 23<br><br>3584 CW Utrecht<br><br>The Netherlands<br><br>Tel. +31-88-7559902<br><br>Fax. +31-30-2516756

PROTOCOL SIGNATURE SHEET

Name	Signature	Date
Prof. Dr. I.M. Hoepelman<br>(Principal Investigator)		

TABLE OF CONTENTS

1. INTRODUCTION AND RATIONALE ... 9
2. OBJECTIVES ... 12
3. STUDY DESIGN ... 13
4. STUDY POPULATION ... 15
4.1 Population (base) ... 15
4.2 Inclusion criteria ... 15
4.3 Exclusion criteria ... 15
4.4 Sample size calculation ... 16
5. TREATMENT OF SUBJECTS ... 16
5.1 Investigational product/treatment ... 16
5.2 Use of co-intervention (if applicable) ... 16
5.3 Escape medication (if applicable) ... 17
6. INVESTIGATIONAL MEDICINAL PRODUCT ... 18
6.1 Name and description of investigational medicinal product ... 18
6.2 Summary of findings from non-clinical studies ... 18
6.3 Summary of findings from clinical studies ... 18
6.4 Summary of known and potential risks and benefits ... 18
6.5 Description and justification of route of administration and dosage ... 18
6.6 Dosages, dosage modifications and method of administration ... 18
6.7 Preparation and labelling of Investigational Medicinal Product ... 18
6.8 Drug accountability ... 18
7. METHODS ... 20
7.1 Study parameters/endpoints ... 20
7.1.1 Main study parameter/endpoint ... 20
7.1.2 Secondary study parameters/endpoints (if applicable) ... 20
7.1.3 Other study parameters (if applicable) ... 20
7.2 Randomisation, blinding and treatment allocation ... 20
7.3 Study procedures ... 20
7.4 Withdrawal of individual subjects ... 26
7.4.1 Specific criteria for withdrawal (if applicable) ... 26
7.5 Replacement of individual subjects after withdrawal ... 26
7.6 Follow-up of subjects withdrawn from treatment ... 26
7.7 Premature termination of the study ... 26
8. SAFETY REPORTING ... 27
8.1 Section 10 WMO event ... 27

8.2 Adverse and serious adverse events 27
8.2.1 Suspected unexpected serious adverse reactions (SUSAR) 28
8.2.2 Annual safety report 28
8.3 Follow-up of adverse events 29
8.4 Data Safety Monitoring Board (DSMB) 29
9. STATISTICAL ANALYSIS 29
9.1 Descriptive statistics 29
9.2 Univariate analysis 29
9.3 Multivariate analysis 29
9.4 Interim analysis (if applicable) 29
10. ETHICAL CONSIDERATIONS 30
10.1 Regulation statement 30
10.2 Recruitment and consent 30
10.3 Objection by minors or incapacitated subjects (if applicable) 30
10.4 Benefits and risks assessment, group relatedness 30
10.5 Compensation for injury 30
10.6 Incentives (if applicable) 31
11. ADMINISTRATIVE ASPECTS AND PUBLICATION 31
11.1 Handling and storage of data and documents 31
11.2 Amendments 32
11.3 Annual progress report 32
11.4 End of study report 32
11.5 Public disclosure and publication policy 29
12. REFERENCES 30

LIST OF ABBREVIATIONS AND RELEVANT DEFINITIONS

ABR	ABR form (General Assessment and Registration form) is the application form that is required for submission to the accredited Ethics Committee (ABR = Algemene Beoordeling en Registratie)
AE	Adverse Event
AR	Adverse Reaction
CA	Competent Authority
CCMO	Central Committee on Research Involving Human Subjects
CV	Curriculum Vitae
DSMB	Data Safety Monitoring Board
EU	European Union
EudraCT	European drug regulatory affairs Clinical Trials GCP Good Clinical Practice
IB	Investigator's Brochure
IC	Informed Consent
IMP	Investigational Medicinal Product
IMPD	Investigational Medicinal Product Dossier
METC	Medical research ethics committee (MREC); in Dutch: medisch ethische toetsing commissie (METC)
(S)AE	Serious Adverse Event
SPC	Summary of Product Characteristics (in Dutch: officiële productinfomatie IB1-tekst)
Sponsor	The sponsor is the party that commissions the organisation or performance of the research, for example a pharmaceutical company, academic hospital, scientific organisation or investigator. A party that provides funding for a study but does not commission it is not regarded as the sponsor, but referred to as a subsidising party.
SUSAR	Suspected Unexpected Serious Adverse Reaction
Wbp	Personal Data Protection Act (in Dutch: Wet Bescherming Persoonsgevens)
WMO	Medical Research Involving Human Subjects Act (Wet Medisch-wetenschappelijk Onderzoek met Mensen

SUMMARY

Rationale: Improving cellular immunity by means of increasing CD4 cells is one of the goals of antiretroviral therapy in HIV, which is achieved by means of virological suppression. A certain group of patients, the so called “immunologic non responders”, fail to reach an acceptable CD4 cell increase despite an adequate virologic response on antiretroviral treatment. Recently a new antiretroviral agent, maraviroc (Celsentry®), is registered for the treatment of patients infected with CCR5 tropic HIV-1 virus. However, data is available suggesting that treatment with maraviroc leads to immune recovery (increase in CD4 cells) in patients who are infected with dual/mixed tropic HIV-1 virus, in the absence of a virologic response. This suggests an alternative mechanism for immune recovery, which could be especially beneficial for this group of patients.

Objective: The primary objective is to confirm the hypothesis that maraviroc stimulates immune recovery; the secondary objective is to explore, by virologic and immunologic investigations, the underlying mechanisms of this hypothesis.

Study design: multicentre, randomized, placebo-controlled, double blind, exploratory mechanistic study.

Study population: HIV-1 infected patients 18 years or older, who meet the inclusion criteria.

Intervention (if applicable): One group receives maraviroc (dose dependent on co-medication), the other group placebo.

Main study parameters/endpoints: A 30% increase in CD4 cell rise in the treatment group (compared with placebo).

Nature and extent of the burden and risks associated with participation, benefit and group relatedness:

1. In the treatment group subjects will start with a registered antiretroviral agent (maraviroc).
2. During the treatment year patients will perform several study visits, probably three more compared with regular visits on the outpatient clinic.
3. Each visit, blood will be drawn by venapuncture for immunologic and virologic investigations (see flow chart).

1. INTRODUCTION AND RATIONALE

The management of patients with HIV infection now allows for the suppression of plasma HIV RNA to ≤50 copies/ml in the majority of antiretroviral therapy (ART)-naive patients within the first 6 months of treatment [1]. Sustained suppression of HIV-1 RNA load associated with the use of "Highly Active Antiretroviral Therapy" (HAART) results in immunologic improvement. In a large prospective cohort of treatment-naive patients, 84% achieved HIV RNA levels of <400 copies/ml within 6 months of starting ART, which corresponded to mean increases in $CD4^+$ T-cell count of 114 cells/µl at 6 months and 181 cells/µl at 12 months [2]. Similarly, a systematic review found mean increases in $CD4^+$ T-cell count of 123 cells/µl at 24 weeks and 160 cells/µl at 48 weeks in treatment-naive patients initiating ART [1]. In patients whose HIV RNA levels are consistently suppressed, $CD4^+$ T-cell counts typically continue to increase for 4 years or more after treatment initiation [3].

Unfortunately, a poor immune reconstitution despite a good virological control (the so called "immunologic non responders") is relatively frequent after initiation of HAART among patients with a baseline CD4 count of <350 cells/microl, older age and the baseline immunological and virological status of the patients.

In the multicenter EuroSIDA study, 29% of patients starting ART at $CD4^+$ T-cell counts <350 cells/µl and in whom HIV RNA load was <500 copies/ml at 6 months had a 'low' $CD4^+$ T-cell increase (defined by an increase in $CD4^+$ T-cell count of <50 cells/µl after 6 months or a similarly low increase up to 12 months) [6]. Likewise, in a multicenter study of subjects starting protease inhibitor (PI)-based therapy for the first time, 17.3% did not achieve an increase of ≥50 $CD4^+$ T-cells/µl at 6 months despite suppression of HIV RNA by at least 1 $\log_{10}$ copies/ml or to <1,000 copies/ml. Such subjects had a higher risk of clinical progression than subjects who had a similar virological response but who did achieve an increase of ≥50 $CD4^+$ T-cells/µl at 6 months [4].

In the Dutch Athena Cohort 17 % out of 6106 patients entered and still on HAART with a suppressed HIV RNA load had a CD4+ count < 200 T-cells/µl at 12 months (F de wolf, personal communication)

A lack of $CD4^+$ T-cell count recovery appears to be the strongest independent predictor of HIV-associated clinical events and death in patients on ART, even when compared with HIV RNA levels [4, 5].

It is now well established that in patients on HAART immune restoration (CD4+ cell increase) is determined by having undetectable HIV viral load. Deeks et al, have shown that this relates to still increased levels of T cell turnover in these patients. It is thought that (as in untreated patients infected with HIV), HIV- induced T cell activation is the driver for first naive and later memory T cell depletion (7-9). Mounting evidence has accumulated from several laboratory that immune activation, either directly by effects of HIV or by bacterial translocation due to HIV, induces massive depletion of CD4+ T memory cells from the gut (10) and gradually causes depletion of CD4 T cells in chronic HIV infection. We have observed that, although prolonged HAART is believed to abolish this systemic immune activation, after the initial steep decline in T cell activation, for years a low degree of activation and expansions of CD8 memory populations can be observed. This may be compatible with ongoing low level viral replication despite the patients having undetectable plasma HIV RNA. As also naive T cell division was observed by Ki67 expression we set out to determine the origins of the naive CD4+ T cells that had been generated in patients on long term HAART. From sophisticated analyses and modeling (using changes in TRECs, Ki67 and telomere length) it appeared that naive T cell restoration was derived from thymus, however also division of peripheral naive T cells contributed (Vrisekoop et al submitted).

Recently a new antiretroviral agent, maraviroc (Celsentry ®), has become available that inhibits HIV-1 infection by selectively binding to the CCR5 coreceptor. In the MOTIVATE 1 and 2 studies performed with maraviroc in antiretroviral experienced patients who were infected with R5 tropic HIV-1 virus, almost a doubling of CD4+ cell count was seen in patients receiving maraviroc compared to the group receiving OBR+ placebo as well as a significant virologic response.
Interestingly however, a significant increase of CD4+ cells was also seen in a randomized trial (Pfizer 1029, evaluating maraviroc vs. placebo in antiretroviral experienced patients with dual tropic (D/M) or indeterminate HIV), despite no difference in virological efficacy between placebo and maraviroc. The pathogenesis underlying this increase in CD4+ cells (despite no virologic effect) in patients infected with dual tropic HIV-1 virus using maraviroc is currently not known. However these data suggests dat other immunological and/or virological mechanisms could be involved in the increase of CD4 cells caused by maraviroc, compared with other antiretroviral agents. Moreover, previous studies investigating other entry inhibitors have indicated that particular amino acid changes in the viral envelope protein are associated with increases in CD4+ T cell count [11].

It is our hypothesis that maraviroc has, by interference with CCR5 activation through its natural ligands or residual HIV, an inhibitory effect on the residual systemic immune activation in patients on HAART which relates to lower CD4+ T cell turnover and increased naive CD4+ T cell gain. This will result in a broader TCR repertoire and better immune competence in the long run.

Hypothesis

Therefore maraviroc, by a yet unknown mechanism, stimulates immune recovery by increasing CD4+ cell count.

2. OBJECTIVES

Primary Objective: to confirm the hypothesis that maraviroc stimulates immune recovery by increasing the CD4+ cell count.

Secondary Objective: to explore, by virologic and immunologic investigations, the underlying mechanisms of this hypothesis.

3. STUDY DESIGN

3.1. General

The MIRS study is a double blind, randomized, placebo-controlled, multicenter exploratory mechanistic study in patients on HAART (in The Netherlands), that after a minimum of 6 months viral suppression (< 50 copies/ml) still have inadequate immune recovery. There will be two study groups (both with undetectable plasma HIV RNA): group one with CD4+ cells < 200 cells per µL and group 2 with CD4 cells between 200-350 CD4+ cells per µL. In both study groups, patients will be randomized to receive either maraviroc or placebo added to the current antiretroviral regimen. After randomization 50% of the patients will receive maraviroc, the remaining 50% placebo. The dose of maraviroc will be adjusted to the co-medication the study subjects are taking (because of farmacokinetic interactions), from 150 mg BID to 600 mg BID according to instructions in the “summary of product characteristics (SPC)” of maraviroc. The duration of study will be one year (48 weeks). Each visit (see below for schedule) blood will be drawn for CD4+ cell count (procedure to be performed in local laboratory at study site). Also, each visit 30 ml of heparin blood en 10 ml of EDTA blood will be drawn for immunologic en virologic studies. All other blood investigations are to be decided on and performed by the treating physician according to standard of care. Furthermore, each visit study subjects will be asked for any adverse events, vital signs will be recorded and physical examination will be performed. At the screening visit and at the end of study visit a complete physical examination will be performed.

3.2 Flow chart

Procedure	Screen	Baseline (day 1)	Week 2, 4, 8, 12, 24, 36 (interim visits)	Week 48 (end of study) or early termination visit	Follow up visit after early termination study (4 weeks)
Randomization		X			
Informed Consent and Eligibility Check	X	X			
Medical History	X				
Physical Exam / Vital Signs / weight	X			X	
Pregnancy test	X				
Targeted Physical Exam / Vital Signs / weight			X		
Adverse Events		X	X	X	
Concomitant Medications	X	X	X	X	X
CD4+ cell count[1]	X	X	X	X	X
Plasma HIV RNA[2]	X		Only: week 4, week 12, week 24 and week 36	X	X
EDTA (virologic studies)[2]	X	X	X	X	X
Heparin blood (immunologic and virologic studies)[3]		X	X	X	X
Dispense Study Medication		X	X		
Collect all study medication				X	

1. CD4+ cell count to be performed in local laboratory at study site

2. 1 tube (each 10 ml) EDTA blood. Plasma HIV RNA will be performed at the site laboratory at screening, week 12, week 24, week 36 and week 48 (or early termination visit).

Depending on local laboratory logistics, in certain sites the 10 ml EDTA blood that will be drawn for virological studies will be partly used for this purpose; in other sites extra EDTA blood (4.5 ml) will have to be drawn at these visits.

3. 3 tubes (each 10 ml) heparin blood

4. STUDY POPULATION

4.1 Population (base)

The populations of interest for this study are those patients infected with HIV-1 in which treatment with HAART did not result in an adequate immunological response despite viral suppression (the so called “immunological non responders”). The study subjects will be recruited from a potential study population of approximately 550 known patients in the Netherlands (A. van Sighem, SHM, personal communication).

4.2 Inclusion criteria

-Age 18 years or older

-HAART with a maximal treatment interruption of two weeks

-viral suppression (< 50 copies/ml) for 6 months

And either:

-CD4+ count < 200 cells/mm3 after minimal one year of treatment with HAART (study group one)

Or:

-a CD4+ cell count between 200 and 350 cells/mm3 after minimal two years of treatment with HAART (studygroup two)

4.3 Exclusion criteria

-Previous use of maraviroc

-HIV-2 infection

-HAART consisting of a combination of tenofovir and didanosine

-Active infection for which antimicrobial treatment

-Acute hepatitis B or C

-Chronic hepatitis B or C for which treatment with (peg)interferon and/or ribavirine

(Note: patients with untreated chronic hepatitis B or C can be included)

-Immunosuppressive medication

-Radiotherapy or chemotherapy in the past 2 years

-Pregnancy or breastfeeding an infant

-Subjects with known hypersensitivity to maraviroc or to peanuts, or any of its excipients or dyes as follows:

- Excipiens from tablet: microcrystalline cellulose, dibasic calcium phosphate (anhydrous), sodium starch glycolate, magnesium stearate.
- Film-coat: [Opadry II Blue (85G20583) contains FD&C blue #2 aluminium lake, soya lecithin, polyethylene glycol (macrogol 3350), polyvinyl alcohol, talc and titanium dioxide.

4.4 Sample size calculation

Sample size calculation was based on an expected difference in rise in CD4 cells of 30% in the treatment group compared with placebo. For this calculation an estimated CD4 cell increase of 10% in the placebo group was used. Estimated standard deviations of CD4 cells are 40% and 30% in the treatment group and placebo group, respectively.

We used an alpha=0.05 and beta=0.10 (power is 0,90). According to this calculation 124 study subjects are needed (62 in the treatment group, 62 in the placebo group). Anticipating a small loss of follow-up, we aim to include 130 patients.

5. TREATMENT OF SUBJECTS

5.1 Investigational product/treatment

The study population will be randomised to receive either the registered, antiretroviral agent maraviroc (a CCR5 inhibitor) or placebo. This will be added to the antiretroviral regimen that the study subject is already taking. Normal dosage is 300 mg BID, but dosage should be adjusted in the case of concomitant use of CYP3A4 inhibitors (150 mg BID) or inducers (600 mg BID). For more details please see Summary of Product Charasterics of maraviroc.

5.2 Use of co-intervention

1. Maraviroc will be added tot the antiretroviral regimen the study subject is already taking.
2. If necessary for clinical reasons, the antiretroviral regimen can be changed at the discretion of the treating physician.
3. Maraviroc is a substrate for CYP3A4 inhibition. The dose of maraviroc has to be adjusted if other medication is co-administered that induces or inhibits CYP3A4 metabolism (see SPC).

4. The concomitant use of maraviroc and St. John`s wort or products that contain St. John`s wort is not recommended (St. John`s wort is expected to decrease maraviroc concentration to suboptimal levels).
5. Patients are advised to use adequate contraception, since there are no data in humans with respect to the potential teratogenicity of maraviroc.

5.3 Escape medication (if applicable)

Not applicable

6. INVESTIGATIONAL MEDICINAL PRODUCT

6.1 Name and description of investigational medicinal product

Maraviroc (generic name maraviroc). See Summary of Product Characteristics (SPC) of maraviroc.

6.2 Summary of findings from non-clinical studies

For product information regarding maraviroc, please SPC.

6.3 Summary of findings from clinical studies

Please see SPC.

6.4 Summary of known and potential risks and benefits

Please see SPC.

6.5 Description and justification of route of administration and dosage

The study medication (maraviroc or placebo) will be supplied as tablets in 150 mg dosage units. Drug supplies will be provided to the central investigator site (pharmacy, department of clinical trial support) as pre-packaged bottles containing 70 tablets per bottle. Subjects will be provided the study medication in sufficient quantities until the next visit.

6.6 Dosages, dosage modifications and method of administration

Maraviroc should be dosed BID, with total dose adjusted according to the other drugs the patient is taking (see SPC). Maraviroc may be taken with or without food. No dose adjustment of other antiretroviral medication the patient is taking is required due to the presence of maraviroc.

6.7 Preparation and labelling of Investigational Medicinal Product

The study medication (maraviroc and placebo) will be prepared, labeled and supplied by Pfizer Inc. This will be done according to relevant GMP guidelines.

6.8 Drug accountability

The study medication will be shipped by Pfizer Inc to the pharmacy (department of clinical trial support) of the central site (UMC Utrecht) and will be stored under recommended storage conditions and in accordance with applicable regulatory requirements.

Each visit, study medication will be dispensed by the pharmacy after a receipt has been prepared by the investigator. Other sites will receive all the necessary medication of each study subject for the entire study from the pharmacy of the central site. The study medication will be stored at the pharmacy of the site and will be dispensed after a receipt has been prepared by the investigator. The investigator will dispose the medication to the study subject. The study subjects will be asked to bring back empty bottles and unused study medication. The number of tablets will be counted and, if more or less than expected, subjects will be asked to account for the difference. Study medication that is left will be destructed by the pharmacy at the site.

7. METHODS

7.1 Study parameters/endpoints

7.1.1 Main study parameter/endpoint

A 30% increase of the amount CD4+ cells in the treatment group compared with the placebo group (for both study groups).

7.1.2 Secondary study parameters/endpoints

-Changes in plasma HIV RNA

7.1.3 Other study parameters (if applicable)

Not applicable.

7.2 Randomisation, blinding and treatment allocation

Randomization to receive either maraviroc or placebo will be done by the pharmacy at the central site. The randomization code will be kept at the pharmacy of the central site. In case of medical safety problems for the patient, the investigator or treating physician can contact the pharmacy of the central site for breaking the randomization code.

7.3 Study procedures

7.3.1 Study visits and procedures

An overview of the schedule of study visits and procedures is presented as a flowchart (see paragraph 3 “study design”).

Study subjects will be asked to visit the outpatient clinic for study visits. There will be nine visits during the study: screenings visit, baseline visit, 6 interim visits and end of study visit. Assuming the patient would perform a visit on the outpatient clinic every three months if he was not participating in this study, this means 5 extra visits to the outpatient clinic. Each study visit CD4+ cell count will be performed in the local laboratory at the study site (2 ml EDTA blood). Also, each visit (except screeningsvisit) 40 ml blood (10 ml EDTA blood and 30 ml heparin blood) will be drawn by venapunction for immunologic and virologic studies. Taken into account that the blood investigations performed as part of this study will partly replace the investigations the treating physician would otherwise anyway perform, this would mean that maximum approximately 322.5 ml blood per study subject will be drawn extra for studypurposes during the study period (48 weeks). If a patient has to be withdrawn from the study and a follow up visit is needed, then an additional 42 ml of

blood will have to be drawn (30 nl for immunological investigations, 10 ml for virological investigations and 2 ml for CD4+ cell count). The blood samples will be transported to the central site (UMC Utrecht) for further analysis. Each visit (except baseline) patients will be questioned for adverse events. Vital signs and weight will be recorded, and, if necessary, targeted physical examination will be performed (to detect changes compared with baseline). Complete physical examination will be performed at baseline and end of study visits. At baseline and interim visits the study medication will be dispensed. Dosing adherence will be assessed (by counting) and drug accountability will be performed at interim and end of study visits

7.3.2 Virological and immunological investigations

7.3.2.1 General

The heparin blood drawn at the study visits will immediately be transported to the department of Immunology of the central site (WKZ, UMC Utrecht) for further analysis. This will be done by a courier under the required storage conditions and regulations. PBMC`s will be isolated and each visit 5 x 10E6 PBMC`s will be sent to the department of virology (Medical Microbiology, UMC Utrecht) for further analysis. The EDTA blood drawn at the study visits will be processed and frozen at the site laboratory. It will be transported to the department of virology of the UMC Utrecht for further analysis.
Plasma HIV RNA will be measured by the site laboratory at the following visits: screening, week 12, week 24, week 36 and week 48 (or early termination visit).

7.3.2.2 Immunologic and virologic investigations

A. To study the mechanism of MVC on the CD4 T cell, we will first address the hypothesis that the CCR5 antagonist has a yet unanticipated effect on CD4+ and not CD8+ T cell homing which may impose a slightly changed distribution between cells in circulation (blood) and tissues. As the bulk of cells (>98) is in the tissues, a minor change in redistribution will have a major effect on CD4 cell numbers in the blood. To analyze this effect in detail, we will phenotype the T cells with well tested naive and memory/effector markers. A rather quick rise in memory cells after start of treatment may point to a redistribution effect, as we have shown after the start of HAART [12].

B. Long term increases in CD4+ T cells could be on the one hand due to naive T cell production from the thymus, even in the elderly, and on the other hand be derived from division of peripheral T cells. As we have shown before in BMT patients and patients on HAART, Ki67, TRECs and telomere analyses can in conjunction with appropriate mathematical modeling discriminate between these two contributions to T cell regeneration. These assays will be performed on viable cryopreserved cells according to standard procedures.

C. To investigate whether the observed gain in CD4+ T cells will have clinical meaning in that the patients have improved immunity to common pathogens (i.e. that the CD4+ T cells are functional) we will perform in vitro functional analyses testing T-cell proliferative and cytokine responses to a set of antigens derived from commonly encountered pathogens (CMV, EBV, Candida). Next to this
classical mitogenic stimuli, like a CD3/CD28 Mab combination and PHA, will be used to test the intrinsic capacities of the T cell compartment.

D. To investigate viral tropism at start of the study, patient PBMCs will be stimulated and co cultivated with MT2 cells to specifically analyze presence of CXCR4 utilizing viruses. Furthermore, the viable viral population as present in longitudinally obtained PBMCs can be quantified by the generation of biological clones on a CCR5 and CXCR4 expressing MT4 T cell line. These biological clones can subsequently be analyzed with respect to polymorphisms in the viral envelope and infectivity of particular CD4+ T cell populations.

E. To investigate whether addition of maraviroc is associated with further reduction in viral production or replication (even below 50 copies/ml), ultra-sensitive plasma HIV-1 RNA quantification (5 copies/ml) will be performed on all longitudinally obtained samples. Furthermore, an ultra-sensitive analysis of the plasma HIV-1 variable part (V3) of the envelope will be performed to investigate (lack of) viral evolution and tropism.

F. Currently, we are setting up a novel diagnostic tool, the Amplicot assay [13] to analyze the diversity of the T cell receptor repertoire. It has been

suggested that the inability of the immune system to respond to a pathogen is caused by the absence of antigen specific T cells, i.e. a to narrow TCR repertoire. With this assay we can test whether the increased CD4 T cell numbers lead to a broader TCR repertoire and improvement of cell immunity.

7.3.3 Investigations using deuterated water in subset of study subjects participating in MIRS

7.3.3.1 Background

We hypothesize that either a low production or a short life span of CD4 cells is causing the so called ‘immunologic non-response’ in certain HIV infected patients treated with HAART. In the ‘Maraviroc Immune Recovery Study’ (MIRS) these patients will be treated either with maraviroc (an new antiretroviral agent) or placebo, to investigate the hypothesis that maraviroc does increase the CD4 cell count in this particular group of patients (due to an immunological mechanism). To further investigate this hypothesis, we plan to do labeling studies with ‘deuterated water’ in these patients. Using this method, it is possible to study the kinetics of CD4 cells, i.e. to measure the production and life span of these cells. This technology is available in the UMC Utrecht in collaboration with the AMC, and has been recently used by us to study (naive) T cell dynamics in controls and treatment naive HIV infected patients (Vrisekoop et al submitted).

7.3.3.2 Objectives

1. Measurement of production of CD4 cells
2. Measurement of life span of newly produced cells
3. To compare variables 1 and 2 in patients receiving maraviroc (verum) with patients receiving placebo.

7.3.3.3 Studypopulation

We aim to include10 study subjects participating in the MIRS study in the UMC Utrecht (no study subjects for labeling studies will be recruited in other centres). No unblinding of the study will be performed. The analysis of the study results of these patients will be descriptive, because due to the size of the study population not enough statistical power is available to perform comparative statistics.

7.3.3.4 Methods

Deuterated water (^2H_2O) is a stable isotope and can be safely used in humans[13, 14] and various studies have been performed using this technique[15]. Study subjects will be asked to drink deuterated water during a period of nine weeks (see flowchart for amount and frequency). During that period and 16 weeks thereafter (total 25 weeks), urine and blood samples will be taken (total 500 ml heparin blood). These blood samples will be taken in addition to those that will be taken for the 'general' MIRS study.

During cell proliferation, deuterium is built in the DNA of new cells. Using the Gas Chromatography and Mass-Spectometry (GCMS) technique, the amount of labeled adenosine can be measured. Subsequently, by mathematical models the kinetics of CD4 cells proliferation and life span can be calculated.

The Deuterated water is manufactured bij Cambridge Isotope Laboratories, Inc (Cambridge, MA, USA). A certificate of analysis can be provided. It will be stored in the pharmacy in accordance with GCP guidelines.

Study subjects meeting the in- and exclusion criteria of the MIRS, will be asked separately if they want to participate in the investigations with deuterated water.

7.3.3.5 Flowchart

**Week**	**1**	**1**	**2**	**3**	**4**
Deuterated water	10 ml/kg (day 1)	1,25 ml/kg (day 2-7)	1,25 ml/kg (day 1-7)	1,25 ml/kg (day 1-7)	1,25 ml/kg (day 1-7)
Blood drawn	50 ml (day 1)		50 ml (day 1)		50 ml (day 1)
urine	X		X		X

**Week**	**5**	**6**	**7**	**8**	**9**
Deuterated water	1,25 ml/kg (day 1-7)	1,25 ml/kg (day 1-7)	1,25 ml/kg (day 1-7)	1,25 ml/kg (day 1-7)	1,25 ml/kg (day 1-7)
Blood drawn		50 ml (day 1)			50 ml (day 1)
urine		X			X

**Week**	**10**	**11**	**12**	**13**	**14**
Blood drawn		50 ml (day 1)		50 ml (day 1)	
urine		X		X	

**Week**	**15**	**16**	**17**	**18**	**19**
Blood drawn	50 ml (day 1)				
urine	X				

**Week**	**20**	**21**	**22**	**23**	**24**
Blood drawn	50 ml (day 1)				
urine	X				

**Week**	**25**
Blood drawn	50 ml (day 1)
urine	X

7.4 Withdrawal of individual subjects

Subjects can leave the study at any time for any reason if they wish to do so without any consequences. The investigator can decide to withdraw a subject from the study for urgent medical reasons.

7.4.1 Specific criteria for withdrawal (if applicable)

If antiretroviral therapy is failing in a study subject (according to the criteria of the national guidelines [14]), the subject will be withdrawn from the study. This study subject will visit the outpatient clinic within two weeks for an 'end of study visit' and 20 ml of EDTA blood will be drawn for further analysis.

7.5 Replacement of individual subjects after withdrawal

Subjects will only be replaced after withdrawal from the study, if necessary for the power of the study.

7.6 Follow-up of subjects withdrawn from treatment

When a study subject has to be withdrawn from the study, an early termination visit will be planned as soon as possible. There will be one follow up visit during which blood will be drawn. This follow up visit will be planned 4 weeks after the early termination visit, or earlier if the patients` antiretroviral regimen will be changed. Further treatment of the study subject will be decided on by the treating physician.

7.7 Premature termination of the study

When unexpectedly, authorities decide for safety reasons to withdraw the registration of maraviroc and hence will be no longer available, the study can be terminated.

8. SAFETY REPORTING

8.1 Section 10 WMO event

In accordance to section 10, subsection 1, of the WMO, the investigator will inform the subjects and the reviewing accredited METC if anything occurs, on the basis of which it appears that the disadvantages of participation may be significantly greater than was foreseen in the research proposal. The study will be suspended pending further review by the accredited METC, except insofar as suspension would jeopardise the subjects' health. The investigator will take care that all subjects are kept informed.

8.2 Adverse and serious adverse events

Adverse events are defined as any undesirable experience occurring to a subject during a clinical trial, whether or not considered related to the investigational drug. All adverse events reported spontaneously by the subject or observed by the investigator or his staff will be recorded.

A serious adverse event is any untoward medical occurrence or effect that at any dose results in death;

- is life threatening (at the time of the event);
- requires hospitalisation or prolongation of existing inpatients' hospitalisation;
- results in persistent or significant disability or incapacity;
- is a congenital anomaly or birth defect;
- is a new event of the trial likely to affect the safety of the subjects, such as an unexpected outcome of an adverse reaction, lack of efficacy of an IMP used for the treatment of a life threatening disease, major safety finding from a newly completed animal study, etc.

All SAEs will be reported to the accredited METC that approved the protocol, according to the requirements of that METC.

Since this is a multicentre study, all serious adverse events will be reported to the central site. The central site will report the serious adverse events to the manufacturer of maraviroc (Pfizer Inc.), using their serious adverse event reporting form for investigator driven research (see addendum).

8.2.1 Suspected unexpected serious adverse reactions (SUSAR)

Adverse reactions are all untoward and unintended responses to an investigational product related to any dose administered.

Unexpected adverse reactions are adverse reactions, of which the nature, or severity, is not consistent with the applicable product information (e.g. Investigator's Brochure for an unapproved IMP or Summary of Product Characteristics (SPC) for an authorised medicinal product).

The sponsor will report expedited the following SUSARs to the METC:

- SUSARs that have arisen in the clinical trial that was assessed by the METC;
- SUSARs that have arisen in other clinical trial of the same sponsor and with the same medicinal product, and that could have consequences for the safety of the subjects involved in the clinical trial that was assessed by the METC.

The remaining SUSARs are recorded in an overview list (line-listing) that will be submitted once every half year to the METC. This line-listing provides an overview of all SUSARs from the study medicine, accompanied by a brief report highlighting the main points of concern.

The sponsor will report expedited all SUSARs to the competent authority, the Medicine Evaluation Board and the competent authorities in other Member States.

The expedited reporting will occur not later than 15 days after the sponsor has first knowledge of the adverse reactions. For fatal or life threatening cases the term will be maximal 7 days for a preliminary report with another 8 days for completion of the report.

8.2.2 Annual safety report

In addition to the expedited reporting of SUSARs, the sponsor will submit, once a year throughout the clinical trial, a safety report to the accredited METC, competent authority, Medicine Evaluation Board and competent authorities of the concerned Member States.

This safety report consists of:

- a list of all suspected (unexpected or expected) serious adverse reactions, along with an aggregated summary table of all reported serious adverse reactions, ordered by organ system, per study;
- a report concerning the safety of the subjects, consisting of a complete safety analysis and an evaluation of the balance between the efficacy and the harmfulness of the medicine under investigation.

8.3 Follow-up of adverse events

All adverse events will be followed until they have abated, or until a stable situation has been reached. Depending on the event, follow up may require additional tests or medical procedures as indicated, and/or referral to the general physician or a medical specialist.

8.4 Data Safety Monitoring Board (DSMB)

Not applicable.

9. STATISTICAL ANALYSIS

9.1 Descriptive statistics

Not applicable.

9.2 Univariate analysis

Not applicable.

9.3 Multivariate analysis

Data will be analyzed according to intention to treat principle. Repeated measures analysis and regression analysis will be performed to detect whether the development over time in CD4 count between the treatment and control group differ significantly.

9.4 Interim analysis (if applicable)

Not applicable.

9.5 Analysis of withdrawn subjects and subjects with missing data

Analysis of subjects who are withdrawn from the study and of subjects with missing data is decribed in the ‘statistical analysis plan’, which is available on request.

10. ETHICAL CONSIDERATIONS

10.1 Regulation statement

The study will be conducted according to ICH GCP guidelines, the principles of the Declaration of Helsinki (adopted by WMA general assembly, Tokyo 2004) and in accordance with the Medical Research Involving Human Subjects Act (WMO) and other guidelines, regulations and Acts.

10.2 Recruitment and consent

Subjects will be informed about the study and asked for their consent by the investigator. They will be given as much time as they need to consider their decision. See also patient information letter and informed consent form.

10.3 Objection by minors or incapacitated subjects (if applicable)

Not applicable.

10.4 Benefits and risks assessment, group relatedness

This study investigates the possible effect of the antiretroviral agent maraviroc (generic name maraviroc) on immune recovery. If the proposed hypothesis is true (a positive effect of maraviroc on immune recovery in immunologic non-responders on HAART) than this would be beneficial for this particular group of patients and could lead to new treatment modalities. Therefore, this study cannot be performed without the participation of subjects who are HIV infected.

10.5 Compensation for injury

The sponsor has a liability insurance which is in accordance with article 7, subsection 6 of the WMO. The insurance of the UMC Utrecht, which has been taken out for this purposes, will cover this.

The sponsor (also) has an insurance which is in accordance with the legal requirements in the Netherlands (Article 7 WMO and the Measure regarding Compulsory Insurance for Clinical Research in Humans of 23th June 2003). This insurance provides cover for damage to research subjects through injury or death caused by the study.

1. € 450.000,-- (i.e. four hundred and fifty thousand Euro) for death or injury for each subject who participates in the Research;

2. € 3.500.000,-- (i.e. three million five hundred thousand Euro) for death or injury for all subjects who participate in the Research;
3. € 5.000.000,-- (i.e. five million Euro) for the total damage incurred by the organisation for all damage disclosed by scientific research for the Sponsor as ‘verrichter’ in the meaning of said Act in each year of insurance coverage.

The insurance applies to the damage that becomes apparent during the study or within 4 years after the end of the study.

10.6 Incentives (if applicable)

Not applicable.

11. ADMINISTRATIVE ASPECTS AND PUBLICATION

11.1 Handling and storage of data and documents

11.1.1 General

Data will be handled anonymously and confidential. Each study subject will receive a randomisation code which will be used to process the data. Only the investigators will have access to the data. Collected data and samples will be stored for 15 years, so that other analysis in the future will be possible.

11.1.2 Monitoring of the study

Monitoring of the study will be performed by qualified monitors of the Julius Center for Health Sciences and Primary Care, UMC Utrecht. A monitoring plan is available on request.

11.1.3 Data management

Data of study subjects will be recorded in a Case Record form (CRF) at the study site. Data from virologic and immunologic analysis performed at the central site will be kept at the central site in a study subject specific file. A database will be constructed for analysis of the data. Statistical analysis will be performed with assistance of a statistician.

11.2 Amendments

A ‘substantial amendment’ is defined as an amendment to the terms of the METC application, or to the protocol or any other supporting documentation, that is likely to affect to a significant degree:

- the safety or physical or mental integrity of the subjects of the trial;
- the scientific value of the trial;
- the conduct or management of the trial; or
- the quality or safety of any intervention used in the trial.

All substantial amendments will be notified to the METC and to the competent authority.

Non-substantial amendments will not be notified to the accredited METC and the competent authority, but will be recorded and filed by the sponsor.

11.3 Annual progress report

The sponsor/investigator will submit a summary of the progress of the trial to the accredited METC once a year. Information will be provided on the date of inclusion of the first subject, numbers of subjects included and numbers of subjects that have completed the trial, serious adverse events/ serious adverse reactions, other problems, and amendments.

11.4 End of study report

The sponsor will notify the accredited METC and the competent authority of the end of the study within a period of 90 days. The end of the study is defined as the last patient’s last visit.

In case the study is ended prematurely, the sponsor will notify the accredited METC and the competent authority within 15 days, including the reasons for the premature termination.

Within one year after the end of the study, the investigator/sponsor will submit a final study report with the results of the study, including any publications/abstracts of the study, to the accredited METC and the Competent Authority.

11.5 Public disclosure and publication policy

The investigator will publish the collected data in medical journals and/or present them on scientific meetings.

12. REFERENCES

1. Bartlett JA, DeMasi R, Quinn J, Moxham C, Rousseau F. Overview of the effectiveness of triple combination therapy in antiretroviral-naive HIV-1 infected adults. AIDS 2001;15:1369-1377.
2. Smith CJ, Sabin CA, Youle MS, et al. Factors influencing increases in CD4 cell counts of HIV-positive persons receiving long-term highly active antiretroviral therapy. J Infect Dis 2004;190:1860-1868.
3. Hunt PW, Deeks SG, Rodriguez B, et al. Continued CD4 cell count increases in HIV-infected adults experiencing 4 years of viral suppression on antiretroviral therapy. AIDS 2003;17:1907-1915.
4. Grabar S, Le Moing V, Goujard C, et al. Clinical outcome of patients with HIV-1 infection according to immunologic and virologic response after 6 months of highly active antiretroviral therapy. Ann Intern Med 2000;133:401-410.
5. Piketty C, Weiss L, Thomas F, Mohamed AS, Belec L, Kazatchkine MD. Long-term clinical outcome of human immunodeficiency virus-infected patients with discordant immunologic and virologic responses to a protease inhibitor-containing regimen. J Infect Dis 2001;183:1328-1335.
6. Florence E, Lundgren J, Dreezen C, et al. Factors associated with a reduced CD4 lymphocyte count response to HAART despite full viral suppression in the EuroSIDA study. HIV Med 2003;4:255-262.
7. Deeks SG, Kitchen CM, Liu L, Guo H, Gascon R, Narváez AB, Hunt P, Martin JN, Kahn JO, Levy J, McGrath MS, Hecht FM. Immune activation set point during early HIV infection predicts subsequent CD4+ T-cell changes independent of viral load. Blood. 2004 Aug 15;104(4):942-7. Epub 2004 Apr 29.
8. Hazenberg MD, Otto SA, Hamann D, Roos MT, Schuitemaker H, de Boer RJ, Miedema F. Depletion of naive CD4 T cells by CXCR4-using HIV-1 variants occurs mainly through increased T-cell death and activation. AIDS. 2003 Jul 4;17(10):1419-24.
9. Hazenberg MD, Hamann D, Schuitemaker H, Miedema F. T cell depletion in HIV-1 infection: how CD4+ T cells go out of stock. Nat Immunol. 2000 Oct;1(4):285-9.
10. Brenchley JM, Price DA, Schacker TW, Asher TE, Silvestri G, Rao S, Kazzaz Z, Bornstein E, Lambotte O, Altmann D, Blazar BR, Rodriguez B, Teixeira-Johnson L, Landay A, Martin JN, Hecht FM, Picker LJ, Lederman MM, Deeks SG, Douek DC. Microbial translocation is a cause of systemic immune activation in chronic HIV infection. Nat Med. 2006 Dec;12(12):1365-71. Epub 2006 Nov 19.
11. Svicher V, Aqaro S, D'Arrigo R et al. Specific enfuvirtide-associated mutational pathways in HIV-1 Gp41 are significantly correlated with an increase in CD4+ cell count, despite virologic failure. J Inf Dis 2008; 197:1408-18.

12. Pakker NG, Notermans DW, de Boer RJ, Roos MT, de Wolf F, Hill A, Leonard JM, Danner SA, Miedema F, Schellekens PT. Biphasic kinetics of peripheral blood T cells after triple combination therapy in HIV-1 infection: a composite of redistribution and proliferation. Nat Med. 1998 Feb;4(2):208-14.

13. Borghans JAM, de Boer RJ. Quantification of T-cell dynamics: from telomeres to DNA labeling. Immunological Reviews 2007; 216 (1), 35–47.

Baum PD, McCune JM. Direct measurement of T-cell receptor repertoire diversity with AmpliCot. Nat Methods. 2006 Nov;3(11):895-901.

14. Peng SK, et al. Arch Pathol 1972 (1) 81-89.

15. Abrams SA, “Stable isotopes in human nutrition”, 2003, CAB international

16. Hellerstein MK. Measurement of T-cell kinetics: recent methodologic advances. Immunol Today. 1999 Oct;20(10):438-41.

17. CBO Richtlijn Antiretrovirale Behandeling 2007. NVAB en NIV**.**
