# Supplementary material for: Maraviroc Intensification of cART in Patients with Suboptimal Immunological Recovery: A 48-Week, Placebo-Controlled Randomized Trial
Source: PLoS One. 2015 Jul 24;10(7):e0132430. doi: 10.1371/journal.pone.0132430 (PMC4514679; doi:10.1371/journal.pone.0132430)
Supplement: S1 Table — (DOCX) [file pone.0132430.s005.docx]

|  | Panel |  |  |  |
| --- | --- | --- | --- | --- |
| 1 | subsets |  |  |  |
| 1 | HLA-DR |  |  |  |
| 2 | CD31 |  |  |  |
| 3 | CCR5 CXCR4 |  |  |  |
| 4 | Annexin-V |  |  |  |
| 5 | Ki-67 |  |  |  |
|  |  |  |  |  |
| Panel | Antigen | Conjugate | Clone | Manufacturer |
| 1, 3, 5 | CD3 | e450 | UCHT 1 | Biolegend |
| 1, 3 | CD4 | APC-Cy7 | RPA-T4 | Biolegend |
| 1, 5 | CD8 | PE | RPA-T8 | Biolegend |
| 1, 2 | CD27 | APC | L128 | BD |
| 1, 2 | CD45RO | PE-Cy7 | UCHL | BD |
| 1 | CD38 | PerCP Cy5.5 | HIT2 | Biolegend |
| 1 | HLA-DR | FITC | LN3 | Ebioscience |
| 2 | CD31 | PE | WM59 | BD |
| 2 | CD3 | FITC | OKT3 | Biolegend |
| 2 | CD4 | Pacific Blue | RPA-T4 | Biolegend |
| 3 | CD8 | PerCP Cy5.5 | RPA-T8 | Biolegend |
| 3 | CCR5 | FITC | 2D7/CCR5 | BD Pharmingen |
| 3 | CXCR4 | PE | 12G5 | BD Pharmingen |
| 4 | CD8 | FITC | G42-8 | BD Pharmingen |
| 4 | Annexin-V | PE |  | BD Pharmingen |
| 4 | 7-AAD | FITC |  | BD Pharmingen |
| 5 | Ki-67 | FITC | MIB-1 | Dako |
| 5 | CD4 | APC-H7 | RPA-T4 | BD Pharmingen |

**S1 Table. Staining panels and antibodies used.**
